# The pathogenic role of intestinal flora metabolites in diabetic nephropathy

**DOI:** 10.3389/fphys.2023.1231621

**Published:** 2023-07-04

**Authors:** En Tian, Feng Wang, Lei Zhao, Yan Sun, Jurong Yang

**Affiliations:** ^1^ The Third Affiliated Hospital of Chongqing Medical University, Chongqing, China; ^2^ Beibei Traditional Chinese Medicine Hospital, Chongqing, China

**Keywords:** diabetic nephropathy, gut microbes, metabolites, novel therapeutics, therapeutic targets

## Abstract

With the increasing incidence of diabetes, diabetic kidney disease has become a major cause of chronic kidney disease. The role of the gut microbiota in diabetes and its related complications have been extensively investigated; the modulatory effect of the gut microbiota on the host depends on several gut microbial metabolites, particularly short-chain fatty acids, secondary bile acids, and trimethylamine N-oxide. In this review, we focused on the evidence related to the pathogenic role of each of the gut microbial metabolites in diabetic nephropathy. The main novel therapies targeting the gut microbiota include probiotics, dietary prebiotics, synbiotic supplements, and faecal microbiota transplants, although there is no standard treatment principle. Further research is therefore needed to elucidate the link between gut microbes and diabetic nephropathy, and more therapeutic targets should be explored to treat diabetic nephropathy with dysbiosis of the gut microbes.

## 1 Introduction

Diabetes mellitus (DM) has become one of the fastest-growing public health problems in both developed and developing countries. According to the 2019 International Diabetes Federation statistics, 463 million people had diabetes in 2019, and 579 million are expected to have diabetes by 2030, and 700 million (51%) by 2045 (Saeedi et al., 2019).

Diabetic nephropathy (DN) is one of the most serious complications of diabetes, and it is also emerging as a major cause of chronic kidney disease (Raval et al., 2020). The pathogenesis of DN is complex and multifactorial, which involves multiple pathways and mediators (Pérez-Morales et al., 2019), including haemodynamic abnormalities, metabolic disturbances and hormone synthesis (Tavafi, 2013). The renin-angiotensin-aldosterone system (RAAS), the formation of advanced glycosylation end products (AGE), activation of transforming growth factor-β1 (TGF-β1), connective tissue growth factor (CTGF), protein kinase C (PKC), mitogen-activated protein kinases (MAPKs) and reactive oxygen species (ROS) are some of the important pathways in the development of DN. Each of these pathways contributes to disease progression through multiple mediators or by interacting with other pathways. For example, oxidative stress causes damages to Ang II, and conversely, RAAS causes damages to oxidative stress; Nicotinamide adenine phosphate dehydrogenase (NADPH) oxidase increases TGF-β, and conversely, TGF-β increases ROS through activation of NADPH oxidase (Samsu, 2021). Among these multifactorial pathogenesis, hyperglycemia is the main cause of DN development (VR et al., 2019). Genetic factors and environmental factors such as diet and lifestyle are widely recognised as contributing to diabetes and its complications, but there is also growing evidence for the involvement of gut microbes (Zhang et al., 2013; Sabatino et al., 2017); endotoxins, proteins, and metabolites from the gut microbiota are associated with kidney disease (Felizardo et al., 2019; Plata et al., 2019; Wu et al., 2020b; Wang et al., 2020), and interventions targeting these causative factors could be potential therapeutic targets to slow the progression of diabetic nephropathy.

## 2 Microbiological characteristics of the gut in diabetic nephropathy

In healthy populations, *the genus Bacteroides* and *phylum thick-walled bacteria* account for more than 90% of the intestinal flora, and the latter includes *the genera Clostridium, Bacteroides, Eubacterium, Prevotella, Porphyromonas, Ruminococcaceae* and *Lactobacillus.* In addition, other species of lower abundance include those belonging to *Proteus species* (*Enterobacteriaceae*, *Helicobacter pylori*, etc.), *Actinobacteria* (*Bifidobacterium* spp. and *Corynebacterium aerogenes*), *Methanococcus,* and *Microphylum* resveratrol (Arumugam et al., 2011). We have counted the intestinal microflora species studied in relation to diabetic nephropathy (Table 1). Some important pathogenic bacteria (e.g., *Klebsiella pneumoniae, Bacteroides immobilis, Enterobacteriaceae* spp.*,* and *Legionella* spp.) proliferate abnormally in end-stage renal disease and DN (Vaziri et al., 2013; Kåhrström et al., 2016). In an animal study, researchers found that the intestinal flora of DN mice showed an abnormal ratio of *thick-walled phylum/Bacteroides*, *that Heterobacteria* and *Bacillus anaerobicus* may contribute to a decrease in glomerular filtration rate, and that *Blautia* spp. may be a protective factor in DN (Li Y. et al., 2020a). In a recent study, Chen et al. (2022) found that the DN group had the highest proportion of the thick-walled phylum, followed by *the genera Bacteroides, Clostridium,* and *Proteus*, whereas the non-DN group had the largest proportion of *the genus Bacteroides,* followed by *thick-walled phylum*, *genus Proteu*s, and *genus Clostridium.* The main differences between the two groups were *Odoribacter splanchnicus, Clostridium cellulolyticum rumen, Kleizzia adler, Lachnospiraceae NK4A136, Ruminococcaceae NK4A214, Aureobasidium* spp.*, Biliophilus* spp, *UBA 1819, Ruminococcaceae UCG-004, Anaerobic* spp.*,* and *Ruminococcaceae NK4A214*. Among them, the *O. splanchnicus*, *Clostridium decidua, Clostridium adelicum,* and *Lachnospiraceae NK4A136 groups* were closely associated with short-chain fatty acid (SCFA) production (Chen et al., 2022). A study confirmed significant differences in the abundance of gut microbiota and changes in bacterial populations in the diabetic group compared to healthy controls and in the DN group compared to the diabetic group. This study also confirmed that *Prevotella* was more abundant in the gut microbiota of healthy controls than in those with diabetes and DN (Tao et al., 2019). The above findings imply that the abundance of certain flora species is higher in patients with DN than in non-DN patients. It is mainly caused by dysbiosis of the intestinal flora, which is manifested by an increase in the species and the number of conditioned pathogens.

**TABLE 1:** Types of intestinal microflora in DN.

Species of flora	Reference
*Klebsiella pneumoniae*	Vaziri et al. (2013), Kåhrström et al. (2016)
*Bacteroides immobilis*	
*Enterobacteriaceae*	
*Legionella*	
*Thick-walled phylum/Bacteroides*	Li et al. (2020a)
*Heterobacteria*	
*Bacillus anaerobicus*	
*Blautia*	
*Thick-walled phylum*	Chen et al. (2022)
*Bacteroides*	
*Clostridium*	
*Proteus*	
*Prevotella*	Tao et al. (2019)
*Oncococcidae*	Cai et al. (2022)
*Sarcococcidae*	
*Streptococcus lactis*	
*Phyla Anaplasma*	Salguero et al. (2019)
*Aspergillus*	
*Verruciformes*	
*Clostridium*	
*Lactobacillus humanus*	Chen et al. (2023)
*Lactobacillus mucilaginous*	
*Fusobacterium*	
*Megasphaeralsdeni*	
*Ruminococcus gnavus*	
*Lactobacillus*	

An increasing number of studies have found that gut microbiota plays a key role in the development of DN (Wu et al., 2020a; Cai et al., 2022). In an animal study, the abundance of gut microbiota changed significantly between the DM and DN groups, particularly the relative abundance of the SCFA-producing bacteria *Oncococcidae, Sarcococcidae,* and *Streptococcus lactis*, and changes in the gut microbiota may play an important role in the progression of DN (Cai et al., 2022). The gut microbiota-derived metabolite phenyl sulfate caused proteinuria and foot cell damage in DN rats (Kikuchi et al., 2019). In a recent study, to assess the effect of gut barrier damage on renal injury in diabetic conditions, investigators induced DN in wild-type and mitochondrial antiviral signalling protein (MAVS)-knockout mice by unilateral nephrectomy and streptozotocin treatment and found that MAVS knockout diabetic mice exhibited more severe glomerular and tubular injury than wild-type diabetic mice. Studies have confirmed the role of MAVS signalling in maintaining the integrity of the intestinal barrier in patients with diabetes (Linh et al., 2022). These findings suggest that dysregulation of the intestinal microbiota is involved in the pathogenesis of DN.

It has been demonstrated that four phyla of lipopolysaccharide-producing Gram-negative bacteria, which includes *the phyla Anaplasma, Aspergillus, Verruciformes*, and *Clostridium*, have been identified as relatively abundant in the gut microbiota of diabetic nephropathy compared to the healthy group (Salguero et al., 2019). By comparing the metabolic profiles of the intestinal flora metabolites of different primary conditions in a study on chronic kidney disease, it was founded that more OTU values were detected in the microbiota of patients with diabetic nephropathy compared to the healthy population and hypertensive renal impairment, in which the relative abundance of *Lactobacillus* hominis and *Lactobacillus* mucosus was higher. *Fusobacterium* spp.*, Megasphaeralsdeni* spp.*, Ruminococcus gnavus* spp. and *Lactobacillus* spp. were also found able to correlate more precisely with L-Proline and Stearic acid were found able to differentiate diabetic nephropathy patients from healthy participants (Chen et al., 2023).

In recent years, host-intestinal flora interactions have become an integral part of host homeostasis. In the context of diabetic nephropathy, there is growing evidence to support a bidirectional microbiota-renal crosstalk, which is particularly evident during progressive renal dysfunction. Indeed, in diabetic nephropathy, the “healthy” microbiota structure is disrupted and gut microbes produce large amounts of uremic solutes, leading to renal damage; on the other hand, the uremic state leads to changes in microbial metabolism and composition due to reduced renal clearance, thus creating a vicious cycle in which In this vicious circle, ecological disorders and renal dysfunction will gradually worsen. Therefore, diabetic nephropathy and intestinal flora are mutually influential and causal.

## 3 The pathogenic role of intestinal flora metabolites in diabetic nephropathy

**FIGURE 1:**
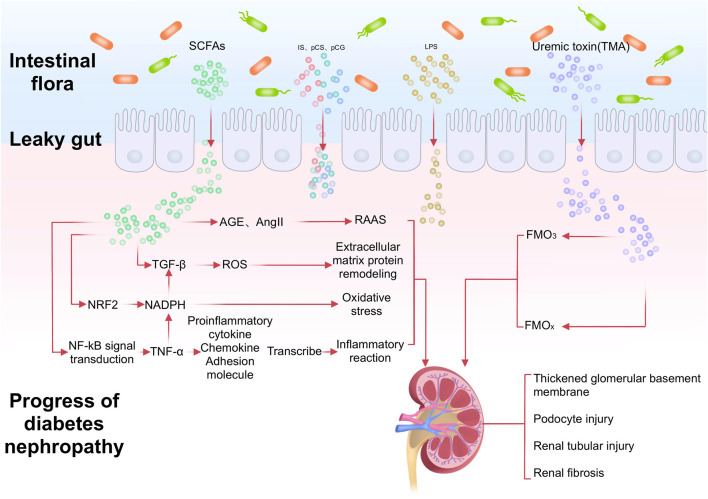
After the imbalance of intestinal flora in DN patients, the production of SCFA decreases and the level of TMAO increases. In addition, the increase of intestinal permeability leads to the increase of LPS permeability. Through different signal pathways, it leads to the thickening of renal basement membrane, podocyte and renal tubule damage, and promotes renal fibrosis.

Intestinal inflammation and epithelial barrier breakdown accelerate systemic translocation of the bacterial-derived uremic toxins including indoxyl sulphate, p-cresyl sulphate, and TMAO, and cause oxi-dative stress injury to the kidney, cardiovascular and endocrine systems (Chen et al., 2019). Glomerular filtration rate decreases with podocyte injury and death, disrupting the homeostasis of the gut microbiota as uremic toxins accumulate in the circulation, i.e., gut microbial dysbiosis (Plata et al., 2019). Subsequently, SCFA production decreases, whereas levels of typical uremic toxins such as p-cresol sulfate, indolyl sulfate, and trimethylamine N-oxide (TMAO) increase, ultimately worsening DN (Feng et al., 2021). In addition, changes in the intestinal microenvironment result in increased intestinal permeability, leading to increased secretion of bacterial components such as lipopolysaccharides (Potrykus et al., 2021). These endotoxins bind to signalling molecules such as toll-like receptors to recruit inflammatory cytokines and contribute to the development of chronic systemic inflammation (Ni et al., 2022) (Figure 1).

### 3.1 Short-chain fatty acids

SCFAs are one of the main metabolites of the microbially-mediated metabolism of fibre in the gut, leading to fermentation (He et al., 2020). It is a subset of fatty acids, containing six or fewer carbon molecules, and shows beneficial effects on the kidney. SCFAs play a role in bioregulation by inhibiting histone deacetylases (HDACs) and activating G protein receptors, thereby reducing the inflammatory response and lowering mean arterial pressure (Magliocca et al., 2022). SCFAs include acetate, propionate, and butyrate, which are produced by microbial communities through the fermentation of indigestible carbohydrates (Cong et al., 2022). Acetate and propionate are mainly produced by Bacteroidetes, whereas butyrate is mainly produced by Firmicutes (Levy et al., 2016).

The SCFA butyrate plays a positive role in the progression of diabetes and certain types of kidney disease (Machado et al., 2012; Guo et al., 2018; Xu et al., 2018), although its mechanism is not yet clear. Butyrate is suggested to alleviate DN by mediating the miR-7a-5p/P311/TGF-β1 pathway (Du et al., 2020). Transforming growth factor-β1 (TGF-β1) is the initial factor that triggers the fibrosis signalling cascade (Chang et al., 2016). P311 is an RNA-binding protein that stimulates TGF-β1 translation in several cell types (Yao et al., 2015). Butyrate supplementation attenuated kidney damage and apoptosis in a mouse model of genetic diabetes (db/db) as well as high glucose-induced fibrosis in SV40-MES-13 cells and inhibited TGF-β1 and P311 expression (Du et al., 2020). Oxidative stress is a key factor in the development of DN (Turkmen, 2017). The nuclear factor erythroid 2-related factor 2 (NRF2) plays a key role in cellular defence against oxidative stress (Kobayashi et al., 2016). Sodium butyrate (NaB), a known activator of NRF2, showed a DN-preventive effect (Zhou et al., 2022). A study that treated streptozotocin-induced diabetic C57BL/6Nrf2-knockout and the corresponding wild-type mice with NaB for 20 weeks found that diabetic mice not treated with NaB showed significant renal pathological changes such as oxidative damage, inflammation, apoptosis, fibrosis, and proteinuria. The results confirmed that NaB inhibited HDAC activity and increased the expression of NRF2 and its downstream targets, heme oxygenase 1 and NAD(P) H dehydrogenase quinone 1. Thus, NaB improved DN by activating NRF2 to inhibit HDAC activity (Dong et al., 2017). A recent study found anti-inflammatory effects of NaB on glomerular thylakoid cells in a mouse model of DN and high glucose-induced mouse glomerular mesangial cells (Gu et al., 2019). Gasdermin D (GSDMD) is a newly identified key executing protein for pyroptosis that is cleaved by inflammatory cysteine aspartase (Deng et al., 2021; Zuo et al., 2021). High glucose was found to increase propidium iodide (PI)-positive cells, promote the release of lactate dehydrogenase (LDH), interleukin-1 *ß* (IL-1β), and release of interleukin-18(IL-18); GSDMD, GSDMD N-terminal structural domain (GSDMD-n), and cleaved protein level caspase-1 levels were also elevated. NaB further enhanced the release of LDH by inhibiting caspase 1-GSDMD, which further enhanced LDH release and the number of PI-positive cells. In addition, NaB or Ac-YVAD-CMK, a caspase inhibitor, reversed the high glucose-induced nuclear factor-κB (NF-κB)/NF-κB inhibitor α (IκB-α) signalling pathway. Thus, NaB ameliorated high glucose-induced pyroptosis in glomerular endothelial cells via the caspase 1-GSDMD classical scorch death pathway involving the NF-κB/IκB-α signalling pathway. Exogenous NaB has been shown in different animal models to improve DN by reducing inflammation and oxidative stress as well as improving fibrosis, cell scorching, and DNA damage (Khan and Jena, 2014; Dong et al., 2017; Xu et al., 2018). Exogenous NaB also protected human glomerular thylakoid cells from high glucose-induced scorch death (Gu et al., 2019). These studies suggest that butyrate may serve as a potential therapeutic target for DN.

Whereas increasing evidence has indicated that butyrate may benefit the host through its anti-diabetic effects, some studies have suggested that acetate may exacerbate disease progression in DN. In a study using diabetic rats as a model, increased serum levels of acetate were found to be associated with increased expression of G protein-coupled receptor 43 (GPR43) in the kidney (Hu et al., 2020). Under high glucose conditions, acetate significantly increased the expression of GPR43 in human kidney 2 (HK-2) cells. Further analysis showed that GPR43 small interfering (si) RNA treatment significantly reduced lipid accumulation in HK-2 cells, even in the presence of acetate plus high glucose, which correlated with reduced protein levels of 3-hydroxy-3-methylglutaryl coenzyme A, low-density lipoprotein receptor, CD36, and chemokine ligand 16. This study confirmed that excess acetate production by the gut microbiota disrupted cholesterol homeostasis through the activation of GPR43, leading to tubulointerstitial damage in patients with DN Another animal study found that diabetic rats had abnormal gut flora, increased plasma acetate levels, increased urinary protein, thickened glomerular basement membranes, and loss of renal peduncle peduncles compared to controls (Lu et al., 2020). In addition, protein levels of angiotensin II, angiotensin-converting enzyme, and angiotensin II type 1 receptor were significantly increased in the kidneys of DM rats. The administration of broad-spectrum antibiotics to DM rats not only completely killed most intestinal flora but also significantly reduced plasma acetic acid levels, inhibited intrarenal activation of the renin-angiotensin (RAS) system (by inhibiting the aforementioned three indicators of RAS activation), and attenuated renal injury. This result suggests that dysregulation of the gut microbiota may be associated with intrarenal RAS activation in early DN. In contrast, plasma acetate levels positively correlated with intrarenal angiotensin II protein expression, inferring that acetate may also be involved in renal injury in early DN (Lu et al., 2020).

Acetic acid, propionic acid, and butyric acid are the main SCFA metabolites produced by gut microbes. However, several studies have focused on butyrate and acetate, and consequently, the potential effects of propionic acid on pathophysiological effects have been underestimated. There is a lack of robust research evidence on the effects of propionate on DN, although researchers have demonstrated that propionate can reduce fatty acid levels in the liver and plasma, reduce food intake, exert immunosuppressive effects, and possibly improve tissue insulin sensitivity (Al-Lahham et al., 2010; Pingitore et al., 2017; Yoshida et al., 2019). Propionate can reduce gluconeogenesis by activating AMP-activated protein kinase (AMPK) to downregulate glucose-6-phosphatase and phosphoenolpyruvate carboxykinase. In addition, siRNA-mediated knockdown of the propionate receptor GPR43 prevented propionate-induced AMPK activation and eliminated gluconeogenesis inhibition (Yoshida et al., 2019). A study group investigating the role of propionate in improving glucose homeostasis *in vivo* and its mechanism of action found that it enhanced glucose-stimulated insulin release and maintained *ß*-cell mass by inhibiting apoptosis (Pingitore et al., 2017). Propionate improves the high-glucose environment in patients with diabetes by modulating insulin sensitivity; thus, it is reasonable to infer that propionate has a positive impact on the progression of diabetic nephropathy.

### 3.2 Trimethylamine N-oxide (TMAO)

TMAO is mainly derived from trimethylamine (TMA) oxidation by intestinal flora. Intestinal microorganisms metabolise and produce TMA from ingested nutrients such as lecithin and choline; these nutrients enter the liver via the portal circulation, where they are oxidised by flavin monooxygenase 3 or other flavin monooxygenases to produce TMAO (Bennett et al., 2013; Koeth et al., 2013). TMAO is a well-known pro-atherogenic sclerosing agent that promotes atherogenesis and is associated with cardiovascular risk (Bennett et al., 2013). Some studies have suggested that gut microbial dysbiosis contributes to diabetic microvascular damage, possibly because of the effects of changes in gut microbiota composition on atherosclerosis and endothelial dysfunction, but there is no conclusive evidence for this correlation (Roberts et al., 2018; Tanase et al., 2020). Evidence indicates that TMAO influences the progression of DN. In an experiment using streptozotocin-induced DN mice as a model, the mice were divided into mild proteinuria (MP) and severe proteinuria (SP) groups. The microbiota composition differed between the two groups, with a decrease in the abundance of thick-walled bacteria and a significant increase in heterobacteria in the SP group, and an increase in thick-walled bacteria in the MP group. This was associated with an increase in blood TMAO levels, which ultimately led to weight gain and increased glucose levels, accelerating the progression of DN. In addition, the lipopolysaccharide produced by the harmful bacteria led to a breakdown of the intestinal barrier, which allowed additional lipopolysaccharide and TMAO to enter the bloodstream and exacerbate renal damage (Li Y. et al., 2020a). In another animal study, TMAO exacerbated renal inflammation and promoted tubulointerstitial damage and renal fibrosis by activating NLRP3 inflammatory vesicles, thereby accelerating the development of DN (Fang et al., 2021b). A recent clinical trial also suggested that alternating gut microflora may be involved in the development of DN and that TMAO and chronic inflammation may be important factors in the development of DN (Yang et al., 2022).

### 3.3 Protein-bound uremic toxins

Protein-bound uremic toxins such as indoxyl sulfate (IS), p-cresol sulfate (pCS), p-toluene glucosinolate (pCG) and phenyl sulfate are derived from the metabolism of aromatic amino acids, tyrosine, phenylalanine and tryptophan by intestinal microorganisms (Fang et al., 2021a). *Escherichia coli, Proteus vulgaris, Paramecium coli, Bacillus liquefaciens*, and *Bacillus mimicus* digest tryptophan to indole and metabolise it to IS in the liver (Keszthelyi et al., 2009). IS directly induces tubulointerstitial injury, oxidative stress and inflammation in post-gonadectomy mice (Ji et al., 2018; Tanaka et al., 2020) and human renal proximal tubular epithelial (HK-2) cells (Ellis et al., 2018), it has also been shown to be associated with the progression of DKD in patients with T1D and T2D, as well as in animal models of diabetes, and its elevation is mainly associated with changes in albuminuria and estimated glomerular filtration rate (Atoh et al., 2009; van der Kloet et al., 2012; Gooding et al., 2019).

Both pCS and pCG are derived from tyrosine, which is converted to p-cresol in the fermentation of *Bacteroidetaceae, Bifidobacteria, Clostridia, Enterococcaceae, Eubacteriaceae, Clostridia, Lachnospiraceae*, and *Lactobacillariidae*, and subsequently combined with sulphate or glucuronide to form pCS or pCG respectively (Russell et al., 2013). pCS can directly affect cell viability and induce cell death as well as induce reactive oxygen species and inflammatory cytokines in HK-2 cells (Watanabe et al., 2013; Park et al., 2019). pCG can cause phenotypic changes in proximal renal tubular cells (Mutsaers et al., 2015). These protein-bound uremic toxins are not only markers for the risk of DN development, but also risk factors that directly contribute to the development of DN. However, the molecular mechanisms of these urotoxins in DN are still unclear and needed further studies.

## 4 Effect of drugs for the treatment of diabetic nephropathy on intestinal flora

For the treatment of DN, glycaemic control is central and the use of hypoglycaemic drugs has been shown to have an impact on the gut microbiota. Metformin is a first-line hypoglycaemic drug that inhibits intestinal glucose uptake and hepatic glycogen output via AMPK-dependent and non-AMPK-dependent pathways and is widely used to reduce hyperglycaemia (Xourgia et al., 2019). Emerging evidence suggests that alterations in gut microbiota are associated with the antidiabetic effects of metformin in mucin-degrading bacteria in patients with type 2 diabetes (de la Cuesta-Zuluaga et al., 2017). An investigator improved glucose intolerance by transplanting gut microbiota from metformin-treated patients into germ-free mice (Wu et al., 2017). In rodent and human studies, metformin has altered diet-related transgenic components, leading to reduced microbial population diversity (Lee and Ko, 2014; Shin et al., 2014; de la Cuesta-Zuluaga et al., 2017).

Another class of hypoglycaemic agents alpha-glucosidase inhibitors, such as acarbose, is an oligosaccharide analogue produced by Acti noplanes, play an important role in reducing postprandial hyperglycaemia by competitively inhibiting alpha-glucosidase activity on the brush border of the small intestinal mucosa, and further reducing the absorption of monosaccharides by the intestinal epithelium (Mushtaq et al., 2023). The reduction in carbohydrate hydrolysis induced by acarbose may have altered microbial fermentation and further reduced lipopolysaccharide and inflammatory cytokines in type 2 diabetes (Su et al., 2015). In an animal study comparing the effects of metformin, acarbose and selegiline on intestinal microorganisms, alpha diversity analysis found that acarbose reduced microbial abundance and diversity. Acarbose selectively increased bacteria including r*umenococci 2* spp. and *bifidobacteria* spp (Zhang et al., 2019).

Glucagon-like peptide-1 (GLP-1) is an enterostatin hormone that delays gastric motility, suppresses appetite, stimulates glucose-dependent insulin secretion and reduces glucagon secretion. Commonly used drugs include DPP-4 inhibitors (e.g., ligliptin, etc.) and GLP-1 receptor agonists (e.g., liraglutide, etc.) (Drucker, 2018). In a study on the effects of sitagliptin on the gut microbiota of rodents, it was found that sitagliptin partially reversed experimentally induced microecological dysbiosis, with an increase in the thick-walled and Tenericutes phylum, a decrease in the anthropoid phylum and a change in the population of SCFA-producing bacteria (Yan et al., 2016). In another animal study it was confirmed that similar results were obtained with vildagliptin treatment (Zhang et al., 2017). In an animal study, acute administration of the glucagon-like peptide-1 receptor agonist (GLP-1RA) liraglutide to mice increased levels of caseinolytic protease B (a component of *E. coli*) and norepinephrine (NE) in the cecum. The results of this study strongly suggest that GLP-1RA causes a surge of *E. coli* due to sympathetic activation receptor agonist on changes in the gut bacterium and the underlying mechanisms (Kato et al., 2021).

Sodium-glucose cotransporter protein (SGLT2) inhibitor that inhibits glucose reabsorption by inhibiting sodium-glucose cotransporter protein 2 in the proximal renal tubule (Heerspink et al., 2016). A study in diabetic mice, in which the gut microbial composition of the subjects was assessed by 16s rRNA sequencing, showed that diabetic mice treated with dagliflozin exhibited a favourable reduction in the ratio of the thick-walled phylum/Bacteroidetes compared to the diet alone group (Lee et al., 2018).

In an animal study, it was explored whether the effects of Sacubitril/Valsartan on DN were related to the gut microbiota and associated plasma metabolic profile. It was found that the Sac/Val intervention partially reshaped the composition of the ancient microbiota, reducing the abundance of some harmful bacteria and increasing the abundance of beneficial bacteria-in particular, the abundance of SCFAs-producing bacteria. This may be one of the mechanisms by which Sac/Val therapy reduces renal fibrosis. Furthermore, through functional prediction analysis, the study verified that Sac/Val not only reshaped the structure of the gut flora in DN patients, but also improved the function of the gut microbiome (Wang et al., 2022).

Although there are more research evidence on strong link between gut microbial dysbiosis and anti-diabetic therapeutic agents, the main effects produced by most antidiabetic drugs are a reduction in the thick-walled phylum/*Bacteroides* phylum ratio and an increase in the metabolites of the gut microbial fraction. However, more studies are needed to confirm the mechanism of action of antidiabetic drugs, especially the newer drugs (DPP-4 inhibitors, GLP-1 receptor agonists, sodium-glucose cotransport protein (SGLT2) inhibitors) on intestinal microbes.

## 5 Interventions for dysbiosis of the intestinal flora in diabetic nephropathy

DN is one of the most serious complications of diabetes and can lead to end-stage renal failure and disability. Changes in the gut microbiota are usually manifested by an increase in the proportion of multiple pathogenic bacteria and a decrease in the proportion of probiotic bacteria (Kikuchi et al., 2019; Tao et al., 2019). Given the serious health implications and economic burden associated with diabetic nephropathy, it is particularly important to find effective interventions to slow its progression. Novel therapies are currently focused on restoring a balanced intestinal environment (ecosystem) using probiotics, dietary prebiotics, and synbiotic supplements (Lopes et al., 2018) (Table 2).

**TABLE 2:** Research evidence on intervention of intestinal flora imbalance in DN.

Type of study	Research object	Interventions	Number of cases	Observation indicators	Reference
Animal experiment	Diabetic Wistar rats	*Lactobacillus*	50	ALT,AST,ALP,TB,γ -GT,Urea, Creatinine,GSH,TG,TL,TC,HDL-C,NF-κB,Bcl-2, Lipid peroxide	Negm El-Dein et al. (2022)
Clinical trial	Diabetes nephropathy patients	Probiotics	76	Fasting blood glucose,2 h postprandial blood glu-cose,glycosylated hemoglobin (HbA1c),microalbuminuria/creatinine (mAlb/Cr) and estimated glomerular filtration rate (eGFR)	Jiang et al. (2021)
Clinical trial	Clinical trial	Probiotic	60	Fasting blood samples, lipid concentrations, biomarkers of inflammation and oxidative stress	Mazruei Arani et al. (2019)
Clinical trial	Diabetes nephropathy patients	Probiotic	60	Glycemic, lipid profiles, biomarkers of inflammation and oxidative stress	Mafi et al. (2018)
Clinical trial	Diabetes nephropathy patients	Probiotic	48	Oxidizedglutathione, total antioxidant capacity, reduced glutathione (GSH), glutathione peroxidase, and glutathione reductase	Miraghajani et al. (2017)
Animal experiment	Male C57BL/6J mice	Probiotic	40	Body weight, glucose homeostasis, adipose tissue inflammation, lipid metabolism, SCFA, intestinal microbiota composition	Alard et al. (2016)
Animal experiment	Male SD rats	Antibiotic	30	Renal Pathological Analysis, Measurement of plasma acetate, 16S rDNA sequencing analysi, Measurement of circulating RAS	Lu et al. (2020)
Animal experiment	Diabetic rats	xylo-oligosaccharides and fructo-oligosaccharides	8	Concentration of lactobacilli、fasting glucose, cholesterol, creatinine, urea, plasma protein, kidney weight, advanced glycationend products	Gobinath et al. (2010)
Clinical trial and animal experiment	DN patients and mice	FMT	180/30	16S rRNA sequencing, random blood glucose, Total urinary protein/urinary creatinine (T/Cr), kidney tissue	Shang et al. (2022)
Research Paper	Human specimens, diabetic mice	FMT	25	BMI, BUN, Scr, TG, TC, HDL-C, LDL-C, UPE, SD, ACR, Microflora, Immunofluorescent staining	Lu et al. (2021)
Animal experiment	Diabetic nephropathy murine	FMT	17	LPS, TMAO, SCRA	Li et al. (2020a)

### 5.1 Probiotics

In a systematic review and meta-analysis, probiotics were shown to have beneficial effects on metabolic indicators in patients with DN, including renal function, glucose homeostasis, lipid metabolism, inflammation, and oxidative stress. Probiotics may be an effective and low-cost treatment for patients with DN (Dai et al., 2022). In a study assessing the effects of oral single-strain or multi-strain probiotic preparations on high-fat diet-induced obesity in mice, the separation between the ability of probiotics to suppress adipose tissue inflammation and limit weight gain was reported. The multi-strain mixture was able to improve fat deposition, insulin resistance, and dyslipidaemia through adipose tissue immune cell remodelling (mainly affecting macrophages). The mixture altered fatty acid uptake at the intestinal level and restored the expression levels of the SCFA receptor GPR43 (Alard et al., 2016). In another clinical trial, it was found that, compared with placebo, 12 weeks after the intake of probiotic supplements, the significantly reduced expression of hs CRP in serum, malondialdehyde (MDA), AGEs, urea, creatinine and IL-1 genes and the significantly increased concentration of glutathione (GSH) in plasma in patients with diabetes nephropathy were related, but did not affect other markers of inflammation and oxidative stress, as well as TNF- α And TGF- *ß* Gene expression (Mafi et al., 2018). Probiotic intake can help reduce inflammatory factors through SCFAs produced by the gut microbes and reduce the production of hydrogen peroxide radicals (March et al., 2017; Lopes et al., 2018). The beneficial effects of probiotics on oxidative damage may be mediated through the production of butyrate in follicles and the reduction of lipid peroxidation (Jiang et al., 2016). In an animal experiment, diabetic rats treated with *Lactobacillus* plantarum or *Lactobacillus* rhamnosus-fermented yoghurt showed the following effects: a significant reduction in blood glucose and α-amylase concentrations; a significant increase in high-density lipoprotein cholesterol concentration and improved lipid distribution; and inhibition of nuclear factor κB and increase in Bcl-2 concentrations (Negm El-Dein et al., 2022). This study conclusively established that the selected *Lactobacillus* spp. had hypoglycaemic potential and could be used as functional nutritional anti-diabetic supplements.

### 5.2 Dietary prebiotic or synbiotic supplements

Of all the exogenous factors affecting the gut microbiome, a long-term diet appears to have, by far, the greatest impact (Xu and Knight, 2015), and it has attracted attention as a therapeutic route to re-establishing a symbiotic relationship. As hyperglycaemia is the main cause of the development of DN (VR et al., 2019), glycaemic control is the cornerstone of DN treatment. Several studies have suggested that dietary prebiotic or symbiotic supplements may modulate glucose metabolism through the modification of intestinal flora, thereby slowing the progression of diabetic complications (Nikbakht et al., 2018; Carvalho et al., 2019; Bock et al., 2021).

There are various types of prebiotics, including oligosaccharides, polysaccharides (e.g., Spirulina, Chlorella, etc.), some natural plant extracts (e.g., vegetables, herbs, wild plants, etc.), protein hydrolysates, polyols, etc. (Yadav et al., 2022). Oligofructose (FOS), a widely commercially available source of prebiotics, has also been shown to improve intestinal disorders and reduce inflammation in patients with diabetes (Pengrattanachot et al., 2022). In animal experiments using rats with type 1 diabetes induced by streptozotocin, FOS showed a protective effect on the kidneys and improvd inflammation and insulin sensitivity (Gobinath et al., 2010). Dietary fibre is a polysaccharide that modulates gut microbiota composition (Cronin et al., 2021). In another animal study, dietary fibre was found to protect mice from the clinical and histological manifestations of DN without altering blood glucose levels. Supplementation with the microbial-derived acetate and butyrate provided similar protection by binding to the metabolite-sensing receptors GPR43 and GPR109A. This study reveals a role for the gut microbiota in attempting to delay the progression of DN through dietary modulation, with SCFA being a key mediator of the protective effect produced by fibre depletion (Li Y. J. et al., 2020b).

### 5.3 Faecal microbiota transplantation

Faecal microbiota transplantation (FMT) may affect gut microbiota composition (Wang et al., 2019). Its success depends largely on the diversity and composition of donor faecal microbes (Zheng et al., 2022). FMT is a new theory and promising technique for the treatment of intestinal microbiome disorders. However, its application is still greatly limited by the risk of disease transmission between donor and recipient, patient acceptance, poor outcomes, and the uncertain impact on the recipient’s immune system (Wang et al., 2019). FMT is a successful treatment for recurrent *Clostridium difficile* infection and is more commonly used in studies of inflammatory bowel disease (Imdad et al., 2018). However, it is now also increasingly being applied in extraintestinal diseases, including haematological disorders, neurological disorders, and metabolic diseases such as diabetes and autoimmune diseases, and has shown some positive results (Wang et al., 2019). In a mouse model of experimental DN, the potential role of gut microbiota was hypothesized to involve renal function regulation, which was validated by the role of the FMT gut microbiome and SCFA produced by it in the treatment of DN (Li Y. et al., 2020a). In addition, Faecalibacterium prausnitzi can be used as a diagnostic and therapeutic biomarker for FMT (Chen et al., 2020; Zhang et al., 2020). The gut structure can be restored by transplanting F. prausnitzii, which could be used as a potential treatment against inflammation and diabetes (Ganesan et al., 2018; Xu et al., 2020). Other studies have also demonstrated that FMT from healthy donors significantly reduced foot cell damage in diabetic rats, thereby delaying kidney damage (Lu et al., 2021).

## 6 Conclusion

There is currently a growing interest in the complex relationship between gut microbes and disease, and an increasing number of researchers are focusing on the area of gut flora and kidney disease, particularly the impact of metabolites of gut microbes on the progression of diabetic nephropathy. We know that the gut microbiota is a regulator of glucose and lipid metabolism and immune inflammation, acting as a link between host and environmental influences. The composition of the gut microbiota of patients with DN differs from that of the healthy population. Studies have demonstrated the relevance of metabolites following gut microbial dysbiosis to the progression of DN. Metabolites such as short-chain fatty acids (SCFA), bile acids (BA) and uremic toxins (TMAO) cause damage to the renal tubules through different signalling pathways, promote renal fibrosis and influence the progression of DN. Although supplementation with probiotics, dietary prebiotics, synbiotic supplements and faecal microbiota transplantation can prevent the progression of DN, improve blood glucose levels, maintain the stability of the body’s environment and reduce the inflammatory response, thus help to improve the quality of life of this group of patients. However, we need to know more about the relationship between gut microbial metabolites and DN to identify additional therapeutic targets to slow the progression of diabetic nephropathy by improving gut microbial dysregulation.
